# Patient radiation safety in the intensive care unit

**DOI:** 10.1093/bjr/tqaf147

**Published:** 2025-07-01

**Authors:** Emilio Quaia

**Affiliations:** Department of Radiology, University of Padova, 35128 Padova, Italy

**Keywords:** radiological imaging, radiation exposure, intensive care unit, justification, optimization, cancer risk, effective dose

## Abstract

The aim of this commentary review was to summarize the main research evidences on radiation exposure and to underline the best clinical and radiological practices to limit radiation exposure in intensive care unit (ICU) patients. Radiological imaging is essential for management of patients in the ICU despite the risk of ionizing radiation exposure in monitoring critically ill patients, especially in those with prolonged hospitalization. In optimizing radiation exposure reduction for ICU patients, multiple parties and professionals must be considered, including hospital management, clinicians, radiographers, and radiologists. Modified diagnostic reference levels for ICU patients, based on UK guidance, may be proposed, especially considering the frequent repetition of X-ray diagnostic procedures in ICU patients. Best practices may reduce radiation exposure in ICU patients with particular emphasis on justification and radiation exposure optimization in conventional radiology, interventional radiology and fluoroscopy, CT, and nuclear medicine. CT contributes most predominately to radiation exposure in ICU patients. Low-dose (<1 mSv in effective dose) or even ultra-low-dose CT protocols, iterative reconstruction algorithms, and artificial intelligence-based innovative dose-reduction strategies could reduce radiation exposure and related oncogenic risks.

## Introduction

Imaging techniques often are employed in intensive care unit (ICU) settings, entailing frequent radiation exposure to monitor critically ill patients and a consequent increase in ionizing radiation exposure with relevant long-term malignancy risk.^1–3^ Although single X-ray imaging procedures involve low doses, repeated chest X-rays (CXRs) and CT scans may result in a high cumulative effective dose (CED, expressed in mSv) in ICU patients, especially in those with a prolonged hospitalization. The largest percentage of radiation exposure, even in ICU patients, comes from CT, nuclear medicine, and fluoroscopy imaging procedures, and a good balance must be found between the increasing clinical demand and ensuring patient safety through exposure justification and optimization. The aim of this commentary review was to summarize the main research evidences on radiation exposure and to underline the best clinical and radiological practices to limit radiation exposure in ICU patients.

## Radiation exposures and risk factors for ICU patients

Many scientific organizations^4–8^ have directed efforts and resources at determining the health effects of radiations and developed different risk models of radiation-induced oncogenic risks mainly derived from the Life Span Study.^9–15^ The oncogenic risk values estimated in ICU patients were found significantly different according to the different oncogenic risk model considered for analysis.^16^ This should imply the need for more reliable and reproducible cancer risk models based on recent epidemiological data in addition the epidemiological data from atomic bomb survivors in the Life Span Study. According to the linear no-threshold (LNT) model^17^ cancer risk caused by ionizing radiation is directly proportional to the amount of radiation exposure. Although other alternative models^18–20^ have been proposed, the LNT remains the most supported oncogenic risk model despite it lacks epidemiological validation and generally overstates the risk of radiation carcinogenesis at low doses.^21–23^

Very different CED values have been reported in many recent studies which have examined radiation exposure risks in ICU patients (Table 1). Leppek et al.^24^ evaluated the CED associated with repeated bedside CXR in ICU patients, particularly those undergoing long-term ventilation for adult respiratory distress syndrome, and found a comparatively mean air kerma value per patient ranging from 0.31 mGy to 0.56 mGy with negligible additional morbidity risk due to CXRs in severely ill long-term ventilated patients. Slovis et al.^25^ focused on radiation exposure from CT scans and the associated attributable risk (LAR). LAR corresponds to the number of excess cancers or deaths due to cancer per 100 000 persons of mixed ages exposed to a radiation dose of 0.1 Gy and is expressed as a percentage (e.g., a LAR of 0.00029 indicates a potential excess of 29 observed cancers in a population of 100 000).^12^ The value represents an approximation of the risk of radiation-induced cancer death, defined as death or incident cases of cancer that would have occurred without radiation exposure but developed at a younger age because of the exposure.^12^ Slovis et al.^25^ also found that the average radiation exposure in ICU patients was 22.2 mSv, with a mean LAR of 0.1% corresponding to a potential 100 observed cancers in a population of 100 000 individuals.

**Table 1.** Cumulative effective dose (CED) range in ICU patients according to the recent literature.

Authors	CED
Leppek et al.^24^	2.49-14.09 mSv^a^
Slovis et al.^25^	2-406 mSv^b^
McEvoy et al.^26^	1-199.89^c^
Moloney et al.^27^	0.04-7.5 mSv^d^
Yee et al.^28^	22.01-48.87 mSv^d^
Krishnan et al.^29^	2-300 mSv^c^
Rohner et al.^30^	2.4-52.1 mSv^e^

a Only X-rays.

b Only CT.

c X-rays, fluoroscopy, and CT.

d X-rays and CT.

e X-rays, CT, fluoroscopy, and nuclear medicine. Values are refereed to one single hospital admission.

McEvoy et al.^26^ assessed CED exposure in ICU patients, considering the medical benefits against associated risks, and found that CXRs contributed to 1.2% of the CED whereas abdominal and pelvic CT scans accounted for 68%. Patients with trauma and longer ICU stays had higher CEDs, but most ICU patients received a lower amount, mainly <1 mSv, suggesting a relatively low risk to patients. Similarly, Moloney et al.^27^ quantified CED from diagnostic imaging performed in ICU patients and found a median CED of 1.5 mSv overall and a median 0.07 mSv CED in the pediatric subgroup. CXR, although the most common modality, accounted for only 2.7% of the total CED, whereas CT scans, used in only 16% of the diagnostic imaging studies, contributed almost 97% of the total CED. Patients with emergent trauma had a significantly higher CED than those with medical or surgical trauma, and length of ICU stay was an independent predictor of a high CED (>15 mSv).

Yee et al.^28^ found that the CED per patient among mechanically ventilated trauma patients in ICU increased over a time frame of 5 years, from 34.59 mSv in 2004 to 40.51in 2009. They attributed this increase to a greater number of CT examinations per patient over the same interval, from an average of 2.11 in 2004 to an average of 2.62 in 2009. Krishnan et al.^29^ observed a CED of ≥50 mSv in 3% of ICU admissions and >100 mSv in 1% of admissions, with a median CED of 0.72 mSv. Higher acute physiologic assessment and chronic health evaluation (i.e., APACHE III) scores, longer ICU stays, sepsis, and gastrointestinal issues all have been associated with higher CED, and CT and interventional radiology procedures have been identified as the most significant contributors to CED. Rohner et al.^30^ discussed safety limits of radiation exposure to minimize the risk of radiation-induced cancer and found that 6.8% of ICU patients experienced doses that were >50 mSv over the effective dose. Similar to a previous study, they also found that higher radiation dose was associated with trauma, extended hospital stays, and more frequent use of CT and fluoroscopy.

## Dose exposure and reduction in ICU patients

Given these increases and the importance of decreasing dose exposure, efforts are ongoing to identify successful strategies. In their retrospective study of patients undergoing CT scans in a medical center that had implemented dose-reduction strategies, Rayo et al. described a 30%–52% decrease in radiation exposure from CT.^31^ Dose-reduction strategies implemented at the center included including low kV, low mAs, use of an effective grid, and automatic exposure control (AEC).^31^ Chan et al.^32^ assessed how implementation of an educational initiative to inform physicians about patient radiation exposure, with a focus on ICU patients with primary neurological disorders, could lower CED in ICU patients. Data on radiation exposure were posted at patient bedsides to determine whether it would influence physician ordering practices. The authors concluded that although estimated effective doses can be reported accurately to physicians, an educational initiative alone is insufficient to alter ordering behaviors. These findings suggest that additional strategies are necessary to mitigate unnecessary radiation exposure in the ICU.

Under the EuroSafe Imaging initiative,^33^ the European Society of Radiology (ESR) and European Federation of Radiographer Societies (EFRS) have made strong commitments to all aspects of radiation protection for patients, including justification, optimization, and dose limitation to as low as reasonably achievable,^6^ and against occupational exposure of staff and exposure of the general population.

**Figure 1.**
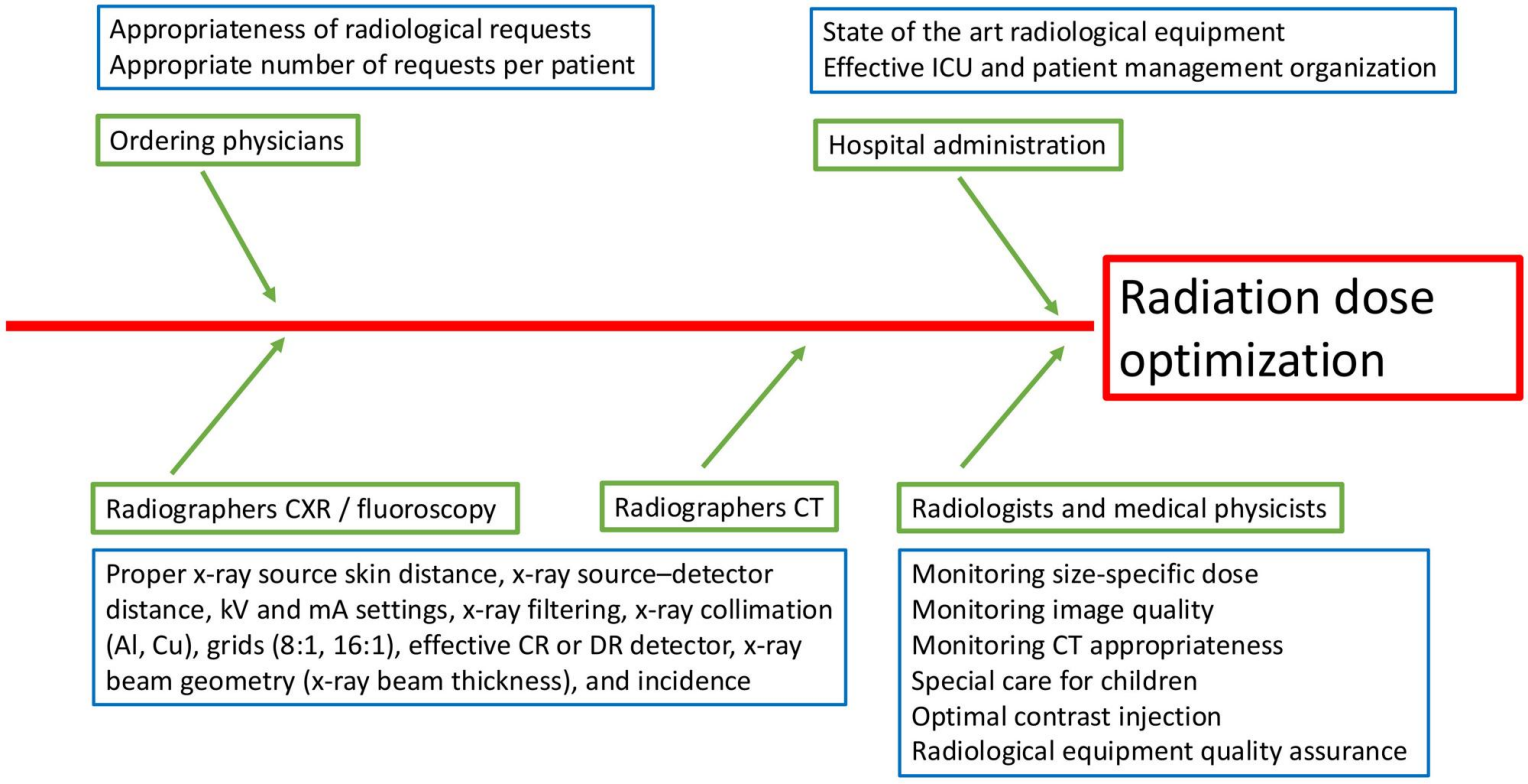
Fish-bone cause-and-effect diagram indicating that multiple parties and actions contribute to achieving radiation dose optimization.

Achieving the desired radiation exposure reduction for ICU patients requires contributions from multiple parties and professionals (Figure 1). The hospital administration should provide state-of-art radiological equipment and space and infrastructure for ICU patient management and hospital ward design, along with appropriate selection and construction of equipment and installations. Clinicians are involved mainly in justification because all individual medical exposures have to be justified in advance according to Euratom/2013/59.^34^ Physicians should send requests to the radiology department, with a limited number of scan repetitions during the hospitalization time frame. Unfortunately, clinicians may not be aware of diagnostic imaging procedure justification principles or have access to a patient’s radiation dose history, and thus, may request already-conducted imaging procedures, including CT, leading to unnecessary patient exposure.^35^^,^^36^ On their side, radiologists should propose educational initiatives for healthcare workers to increase awareness about radiation risks and promote safer practices, including limitation of repeated CXRs, fluoroscopy, and CT scans, use of real-time dose monitoring systems, and a generally more balanced approach to radiological imaging in the ICU, stressing minimized radiation exposure while ensuring continuity of high-quality medical care.

Radiologists and radiographers are mainly involved in optimization and dose limitation in ICU patients, but medical physicists must assure proper quality assurance in radiological equipment with optimization of radiation exposure during each examination (Figure 1). According to the International Commission on Radiological Protection (ICRP), optimization is the process of adjusting protection measures for a source of radiation to maximize the net benefit and minimize risk. The type of imaging procedure, dose, and other technical parameters (kV, mAs, beam collimation) must be adapted to the specific clinical question.^37^ To support optimization and dose limitation, the Council Directive 2013/59/Euratom Basic Safety Standard^34^ requires the establishment, regular review, and use of diagnostic reference levels (DRLs) set as the 75th percentile of distributions of measured dose values for each imaging technique, obtained in a sample of representative national centers.^37–39^ DRLs are indicators for the typical practice in a country or in a region and are related to the equipment and procedural protocols employed. DRLs are not intended for application to individual patients, as body mass and habitus can vary and some patients may require a higher (or lower) dose than a set standard.

DRL measurements include dose-area product (DAP; also called the air kerma-area product or PKA), which corresponds to the dose × beam area; the entrance skin dose (ESD), which corresponds to entrance surface air kerma (Ka, e) in conventional X-rays; DAP and fluoroscopy time, both for fluoroscopy and interventional radiology procedures; and volume CT dose index (CTDI_vol_) and dose length product (DLP) for CT.^40^ To account for variations in patient body size, the American Association of Physicists in Medicine has introduced size-specific dose estimates (SSDEs),^41^ which correspond to CTDI_vol_ × a conversion factor (*f*_size_, estimated using software or users to measure patient diameter—anteroposterior, lateral, or both) to normalize CTDI_vol_ to patient size. The effective dose is estimated by multiplying DAP or DLP by specific conversion factors.^42^ Unfortunately, no conversion factor is available to estimate an effective dose from SSDE.

Radiation dose monitoring systems have been implemented in many medical imaging departments to provide valuable information concerning justification, optimization, and dose limitation with different modalities^43–45^ and represent a further useful tool for limiting dose exposure in ICU patients especially for those with a prolonged hospitalization time. To calculate the effective dose based on Monte Carlo simulations, radiation dose monitoring systems extract patient sex, age, date and time of the examination, scan region, study description, protocol name, scanner manufacturer and model, and dose metrics from a radiation dose structured report sent to the picture archiving and communication system (PACS).

In contrast to occupational dose limits, DRLs were proposed to be applied to the general population undergoing diagnostic X-ray procedures and may be exceeded if required according to the level of clinical gravity and emergency as in the case of ICU patients. Conversely, considering the frequent repetition of X-ray diagnostic imaging examinations in ICU patients a general reduction of DRL assigned to each radiological examination to reduce the whole radiation exposure and CED should be considered.

According to these observations, specific DRL for ICU patients can be proposed based on the recent literature exposure values^24–30^ (Table 1) and on the UK National Diagnostic Reference Levels guidance from the UK Health Security Agency^38^ and ICRP recommendations^39^ (Tables 2 and 3).

**Table 2.** Diagnostic reference levels per exam for the most common X-ray procedures in ICU patients.^a^

Technique	DAP (Gy cm^2^)	ESD (mGy)
Chest radiography AP	0.15	0.2
Chest radiography LL	0.15	0.5
Abdomen radiography	2.5	4
Interventional fluoroscopy (30 min)	500	3000

a Values based on UK National Diagnostic Reference Levels guidance from the UK Health Security Agency,^38^ modified for ICU patients. Considering the frequent repetition of x-ray diagnostic imaging examinations in ICU patients, specific diagnostic reference level (DRL) values for different imaging techniques should be proposed for ICU patients. Table shows desirable DLR value for the different imaging techniques employed in ICU patients and according to the reference values reported in recent studies.

**Table 3.** Diagnostic reference levels per exam for CT in ICU patients.^a^

Technique	CTDI_vol_	SSDE	DLP
	(mGy)	(mGy)	(mGy cm)
CT head	40	120	500
CT chest	8	8	200
CT chest high resolution	8	8	250
CT pulmonary angiography	9	9	250
CT coronary angiography (prospective)	9	9	150
CT coronary angiography (retrospective)	10	10	350
CT chest and abdomen	10	10	300
CT abdomen and pelvis	10	10	300

Abbreviations: AP = anteroposterior; CTDI_vol_ = volume computed tomography dose index; DAP = dose-area product, also called kerma-area product; DLP = dose length product; ESD = entrance skin dose, also called entrance surface air kerma; LL = latero-lateral; SSDE = size-specific dose estimate.

a Values based on UK National Diagnostic Reference Levels guidance from the UK Health Security Agency^38^ modified for ICU patients. Considering the frequent repetition of X-ray diagnostic imaging examinations in ICU patients, specific diagnostic reference level (DRL) values for different imaging techniques should be proposed for ICU patients. Table shows desirable DLR value for the different imaging techniques employed in ICU patients and according to the reference values reported in recent studies. DLP is higher in high resolution chest CT than in conventional chest CT due to higher mAs value as in UK Health Security Agency.^38^

## Best practices for radiation exposure reduction in ICU

Some best practices for radiation exposure reduction in ICU patients should extensively be applied, as detailed below. Healthcare professionals should consistently apply these best practices to minimize radiation exposure without compromising diagnostic quality although daily routine and consolidated habits could hamper their consistent application. Moreover, the ordering behaviors from clinicians are difficult to be modified both due to the routine clinical habits and even to clinical guidelines which not always addressed completely radioprotection principles. Clinical guidelines sometimes do not fully incorporate radioprotection principles, particularly the ALARA principle. While guidelines may outline best practices for various imaging procedures, they might not always explicitly address the need to optimize dose while maintaining diagnostic quality. This can lead to unnecessary radiation exposure, especially in ICU patients and in those diagnostic procedures where the benefits of diagnostic imaging are less clear or when alternative imaging modalities are available.

### Portable CXR

CXR is the most commonly requested diagnostic imaging examination in ICU patients.^46^^,^^47^ Ordering routine daily CXRs is fairly common in ICU patients and bedside CXRs are frequently overprescribed in ICU patients. Despite bedside CXRs are helpful in evaluating the chest in critically ill patients, routine daily CXRs do not necessarily improve patient outcomes and may even lead to unjustified increased radiation exposure.^24–31^ A reduction in the number of CXRs during hospitalization could represent a first strategy in reducing radiation exposure.^47–49^ Routine CXRs often show clinically significant or even unexpected abnormalities in this patient population, many of which can determine significant changes in clinical management. However, after the initial imaging, systematic daily routine CXRs can likely be eliminated without increasing adverse outcomes in adult ICU patients.^49^ Clinicians should ensure that imaging procedures are really clinically necessary and that the number of CXRs taken is minimized.

To maximize image quality, CXR in the ICU is generally obtained with the patient supine and with the cassette in the vertical dimension to include the upper airway and upper abdomen and allow for evaluation of endotracheal tubes, feeding tubes, subpulmonic pneumothorax, and pneumoperitoneum, among other important findings. Clips, telemetry wires, and other external objects should be removed from the field as much as possible to better identify the position of lines, tubes, and the subtle findings of pneumothorax and early consolidation. The need to obtain adequate penetration to see the line and catheter positions necessitates increasing kV, leading to reduced image contrast and exposure time, thus, increasing motion artifacts. In addition to increasing radiation exposure, these practices cause deterioration of the X-ray image, often yielding a film that is suboptimal for evaluating subtle changes in lung parenchyma. Reduced exposure time with well-penetrated CXRs through proper kilovoltage can be obtained.

**Figure 2.**
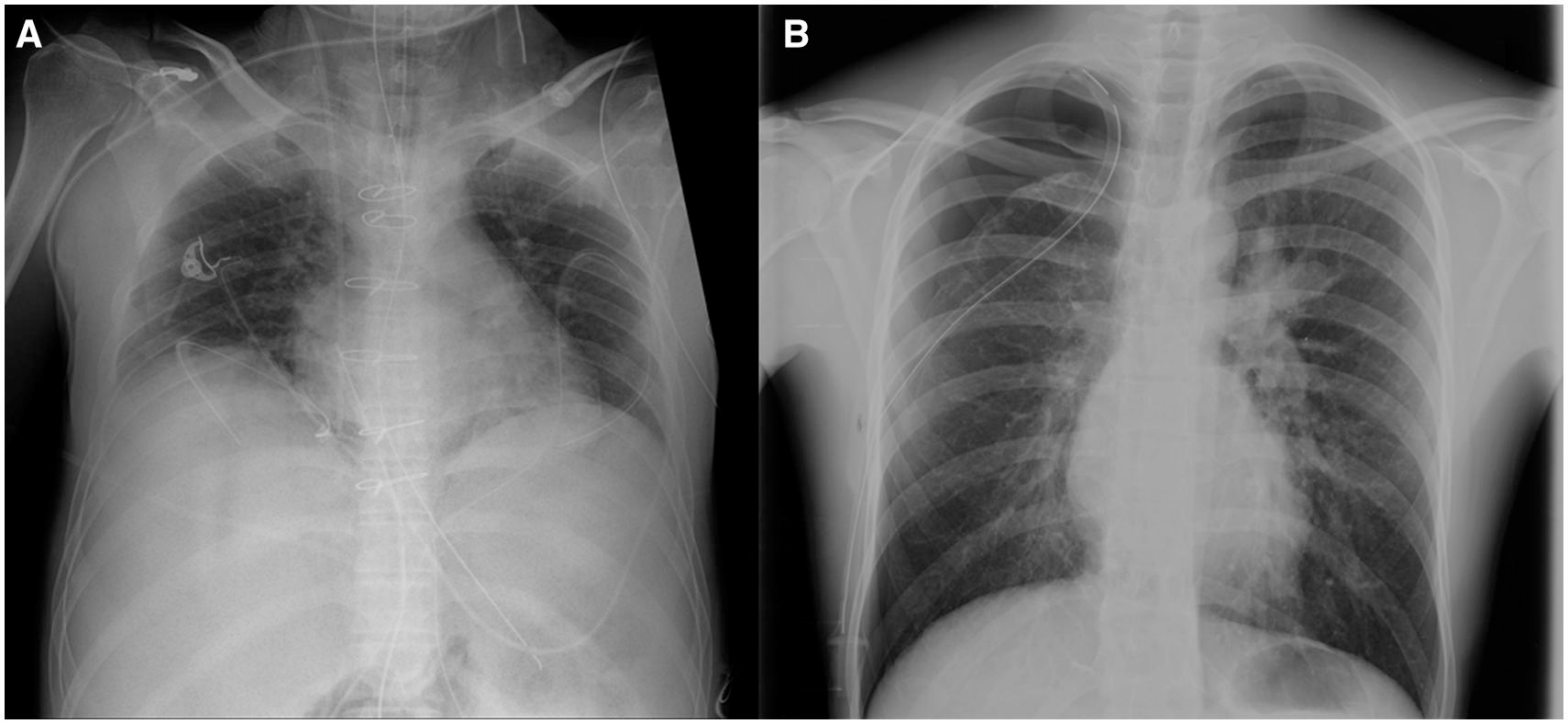
Chest X-rays of ICU patients. (A) Chest X-ray acquired using improper X-ray beam collimation, as the upper abdomen is included, with a consequent increase in dose–area product and effective dose. Multiple devices are present, including a central venous catheter, tracheal tube, nasogastric tube, and chest tubes. (B) X-ray beam collimation was appropriately limited in this patient, as only the chest region is included. A pneumothorax is visible on the right apex, drained by a chest tube.

Best practices to limit radiation exposure from CXR in ICU patients while preserving image quality may include use of modern X-ray mobile systems with digital radiography or computed radiography detectors with high quantum efficiency; adaptation of protocol parameters (e.g., X-ray source–skin distance [SSD] of 120 cm, X-ray source–detector distance, kV [60-100 kV] according to patient size, mA [1-10 mA] and mAs product, X-ray filtering with a 0.1-mm copper filter); accurate patient centering and X-ray collimation (Figure 2); grids (8:1, 16:1); and X-ray geometric incidence to the patient with a limited body region exposed to X-ray radiation. The addition of an automatic exposure device additionally enhances quality and allows for more consistent lung density between serial examinations, especially in patients with rapid fluid shifts between different compartments. Ultrasound can be an important, radiation-free alternative to CXR to assess cardiac function, investigate the source of thrombotic embolism, assess pleural effusion and abdominal collections, assess the lung parenchyma, and guide invasive procedures. Ultrasound scanning of the lung shows a high diagnostic accuracy for the most common etiologies of respiratory failure, such as pneumothorax, pulmonary edema, pneumonia, and pleural effusion. In critical care medicine, ultrasound has demonstrated better diagnostic sensitivity for respiratory pathologies than CXR.^50–54^

### Interventional radiology and fluoroscopy

ICU patients, particularly those undergoing multiple fluoroscopic procedures, are at risk of higher radiation exposure compared to other hospitalized patients. Fluoroscopy, in particular, can lead to elevated doses, especially during prolonged interventional procedure.^55^^,^^56^ Fluoroscopy is mainly used for interventional purposes or to evaluate the upper gastrointestinal tract after surgery in ICU patients. The varying complexity of pathologies treated using fluoroscopically guided endovascular approaches leads to a significant number of prolonged procedures, resulting in increased radiation exposure to patients and even operators. The radiation doses among fluoroscopically guided procedures can vary significantly, and radiation dose displays have been required on fluoroscopy screens to reduce patient radiation exposure.^55^ Best practices to reduce exposure dose in fluoroscopy should include ensuring an appropriate X-ray SSD and X-ray source–detector distance, kV of 60–120 kV and mA of 1-10 mA, X-ray filtering, X-ray collimation (Al, Cu), grids (8:1, 16:1), an effective detector, automatic brightness control, limited pulses per second (30, 15, 7.5, 3.75 frames/s), limited fluoroscopy time, and reduced magnification, X-ray beam geometry (X-ray beam thickness), and incidence to the patient. Recent guidelines have defined a significant radiation dose threshold as any of the following: fluoroscopy time <60 min, ESD <3000 mGy, and DAP <500 Gy·cm^2^.^56^

### CT

The present overexposure of ICU patients to diagnostic X-rays mainly comes from CT overuse. While CT scans are valuable diagnostic tools, their overuse can lead to increased radiation exposure and potential health risks, according to various sources. ICU patients can be commonly exposed to radiation doses >100 mSv during a single hospital admission, particularly with prolonged hospitalization.^27^ CT imaging is often compromised by low image quality and artifacts due to external or internal medical devices and patient arms placed along the body. ICU patients are frequently transported to the CT unit despite labile circulatory function and being connected to monitors, ventilators, and various lines, catheters, and surgical appliances. Portable CT scanners for head CT could improve ICU patient management as patients often are deemed too unstable for transport to a CT scanning room.^27^^,^^57^ Although these scanners are not yet available in every hospital, there is potential for further developments in portable CT technology with further scanner size reductions and image quality improvement.

Unfortunately, CT justification practices vary across Europe, with less than 50% of countries using advance justification^58^ even as justification practices should be much more extensive, especially in ICU patients. Regarding optimization, specific emphasis should be placed on dose-reduction methods, such as low-dose CT (<1 mSv) scanning. To limit radiation exposure, low-dose^59^ and ultra-low-dose CT^60^ offering a sub-millisievert radiation should be considered routinely in ICU patients, especially if they are scanned several times during a hospitalization, provided that the necessary diagnostic content is maintained. Presently, ultra-low-dose CT protocols are increasingly used in clinical practice, which should be especially emphasized in ICU patients, particularly with chest and spine CT and the high intrinsic contrast between structures, excluding overweight patients as noise levels are too high to maintain the diagnostic content of CT images.

CT scanning parameters-including tube current (mA), tube current–time product (mAs), tube rotation time/exposure time (s), tube peak voltage (kVp), collimation, filters, table feed, pitch, and automatic exposure control–all closely influence CTDI_vol_ and radiation effective dose during the acquisition phase and should be optimized. Optimization of applied tube current is the most commonly used method for adjusting radiation dose, which can be achieved with either fixed tube current or automatic AEC applied angularly and/or longitudinally to maintain the preselected image quality based on noise index or standard deviation. Although AEC may enable substantial radiation dose reduction in comparison to fixed tube current, particularly in smaller and average-sized patients, it can actually necessitate in an increased dose to obtain the desired image quality in larger patients. Tube peak voltage presents an exponential relationship with dose, ranging from 2.5 to 3.1, depending on patient size. Although 3-4 fixed presets of tube potential are usually available in most CT scanners (typically 80, 100, 120, and 140 kVp), modern CT scanners offer up to eight presets (70, 80, 90, 100, 110, 120, 130, 140, and 150 kVp). The advantage of kVp in comparison to mAs is that noise increase related to the kVp decrease is compensated by higher contrast even if patient size still represents a limitation in kVp reduction. Tin filter can provide effective X-ray beam hardening and dose reduction.^61^

Further advances in technology, including improvements in AEC and beam-shaping bow-tie filters, could allow further exposure dose reduction^47^^,^  ^59–61^ in ICU patients. An easy way to avoid excessive radiation dose is to select the appropriate anatomic coverage based on easily identifiable landmarks on a planning radiograph with the aim of reducing DLP. In addition to radiation exposure in CT, a focus on iodinated contrast injection in ICU patients is needed, specifically in high-risk cohorts, to evaluate the risk of post-contrast acute kidney injury among patients with a higher burden of acute and chronic medical conditions and clinical risk factors, particularly with a glomerular filtration rate <45 mL/min/1.73 m^2^.^62^

**Figure 3.**
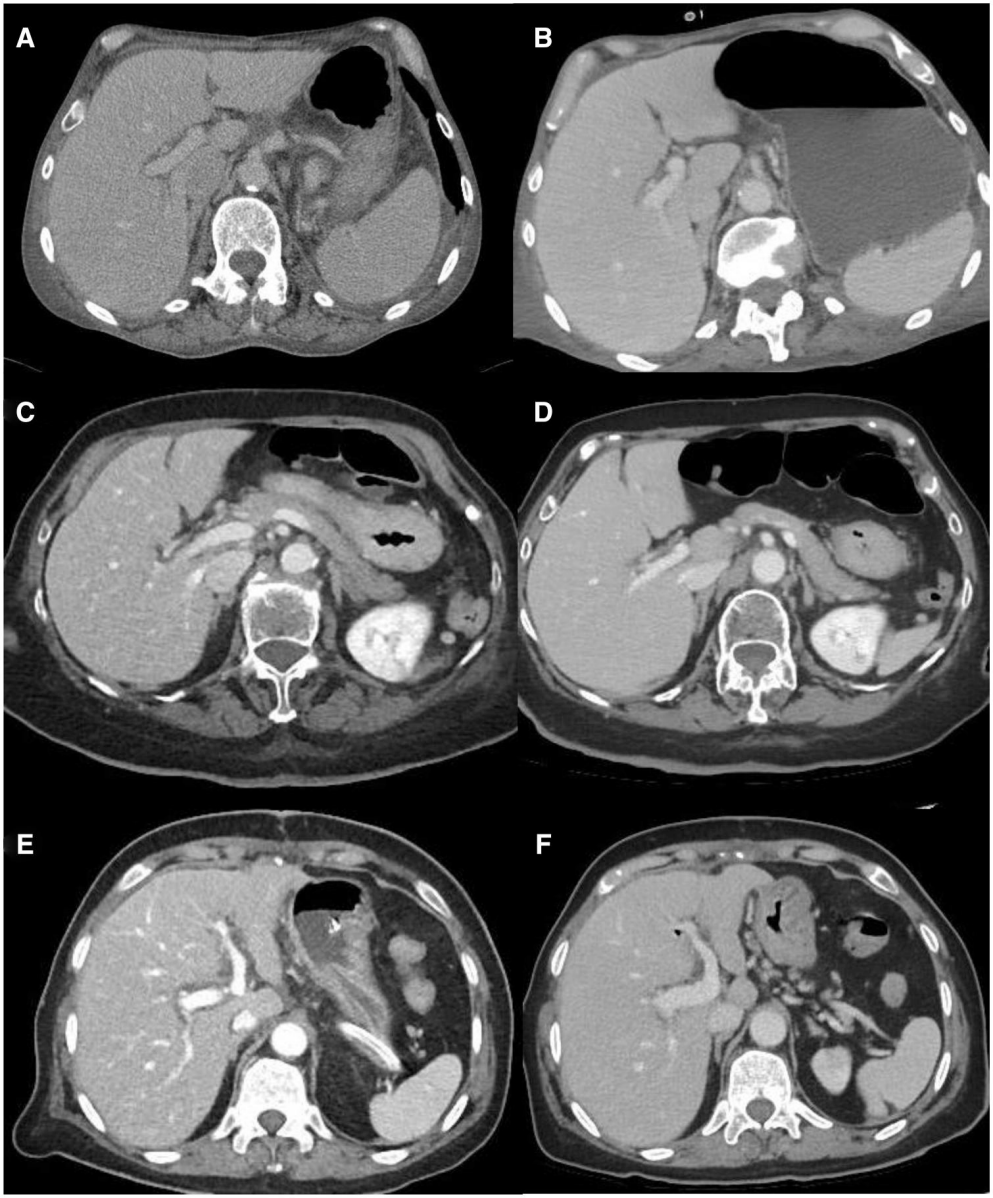
Visual differences in abdominal parenchyma border definition and noise among different reconstruction algorithms. (A, B) 45-year-old male patient after major surgery. (C, D) 47-year-old male patient after lung transplant. (E, F) 55-year-old male patient after cardiac transplant. Images produced using different models were scored by reviewers, as follows: Filtered back projection (FBP) (A), advanced modeled iterative reconstruction model-based iterative reconstruction (ADMIRE) (C), and adaptive iterative dose reduction 3D (AIDR 3D) hybrid iterative reconstruction (E) vs. deep learning image reconstruction (DLIR) (B, D, F). From Quaia et al.^65^

Along with technical parameters to set before ICU patient scanning, iterative reconstruction (IR) algorithms represent another effective tool to reduce ICU patient CT-related exposure dose (Table 4). In hybrid statistical IR, the sinograms are iteratively filtered to reduce artifacts and image noise and backward-projected into the image domain. In model-based IR artificial sinograms are compared with true sinograms to optimize images and remove noise in three to five iterations. The main strengths of IR methods are (1) dose reduction by reconstruction of low-noise image data from intrinsically noisy reduced-dose CT acquisitions and (2) reduced CT image artifacts resulting from a non-true representation of what should be present in the real image corresponding to noise and artifacts.^63^^,^^64^ Deep neural network (DNN)-based deep learning image reconstruction (DLIR) represents a further effective tool to reduce radiation exposure. The DLIR engine generates the output image from an input sinogram acquired at a low radiation dose by employing DNN-based models. During training, DNNs analyze the data and synthesize a reconstruction function, which is optimized through the learning process and extensive testing of a dataset for validation (Table 4). DLIR reconstruction involves three modes (DLIR-Low, Medium, and High), and the final output image is generated by varying the degree of noise included in the image for each mode. DLIR provides a marked reduction in radiation effective dose in ICU patients^65^ and improves the signal-to-noise ratio compared to both the filtered back projection (FBP) and IR algorithms (Figure 3), even with a reduced radiation dose.^65^ Model-based IR and DLIR algorithms are more computationally demanding, resulting in notably increased reconstruction times that potentially could limit their use in clinical practice. Reconstruction time for DLIR is three to five times shorter for MBIR. It is reasonable to expect a wider implementation of DLIR algorithms in the future given the increasing computational power of CT scanners. Further evaluation is needed, however, to investigate whether the improved image quality with DLIR affects subjective CT image quality, CT workflow, and efficiency in terms of the time needed to assess CT images.

**Table 4.** Different reconstruction algorithms from the major vendors.

Vendor	Algorithm name	Type of algorithm	Reconstruction speed	Artifact reduction	Noise reduction
GE Healthcare	ASIR	Hybrid IR	+	+	++
	ASIR-V	Hybrid IR	+	+	++
	Veo	Model-based IR	–	++	+++
	TrueFidelity ^a^	DLIR	+	+++	++++
Philips Healthcare	iDose	Hybrid IR	+	+	++
	IMR	Model-based IR	–	++	+++
	Precise Image^b^	DLIR	–	+++	++++
Siemens Healthineers	IRIS	Image space IR	+++	–	+
	SAFIRE	Hybrid IR	+	+	++
	ADMIRE	Model-based IR	+	++	+++
Canon Healthcare	AIDR3D	Hybrid IR	+	+	++
	FIRST	Model-based IR	–	++	+++
	AiCE^c^	DLIR	+	+++	++++

TrueFidelity and Precise Image are direct DLIR algorithms. AiCE is an indirect DLIR algorithm reconstructing the sinogram directly into an image, without the use of filtered back projection or iterative algorithms. − minimal; + average; ++ fast/strong; +++ very strong; ++++ extremely strong.

Abbreviations: ADMIRE = Advanced Modeled Iterative Reconstruction; AiCE = Advanced Intelligent Clear-IQ Engine; AID3D = Adaptive Iterative Dose Reduction 3D; ASIR = Adaptive Statistical Iterative Reconstruction; DLIR = deep learning image reconstruction algorithm; FIRST = Forward projected model-based Iterative Reconstruction Solution; IMR = iterative model reconstruction; IRIS = iterative reconstruction in image space; SAFIRE = Sinogram-Affirmed Iterative Reconstruction.

a Trained by high-quality filtered back projection datasets acquired in both phantoms and patients as ground truth.

b Trained by lower-dose-simulated sinograms (input) and matched routine-dose filtered back projection images as ground truth.

c Trained by patient lower-dose HIR images as input and routine-dose full MBIR images as ground truth.

Novel imaging technology photon-counting CT scanners can further reduce radiation doses and improve image quality based on detector quantum efficiency. These scanners allow for better signal-to-noise ratios for the same dose, especially in overweight patients, resulting in images of comparable quality to traditional CT.^66^ The implementation of dose-tracking software and real-time dose monitoring provides immediate feedback, facilitating dose optimization.

### Nuclear medicine

Nuclear medicine techniques are frequently employed in ICU patients and in critical patients. Common applications include pulmonary embolism detection by pulmonary scintigraphy and evaluation of patients with post biliary tract surgical complications by hepatobiliary scintigraphy.^67^ The extended use of hybrid imaging, such as PET-CT, entails significant radiation exposure. The features of the radioisotope (half-life, type of radiation emission), as well as the characteristics of the administered pharmaceutical (organ uptake distribution, elimination pathways, biologic half-life), all influence patient radiation exposure. Isotopes with a physical half-life in the range of the total examination duration should be applied to prevent unnecessary radiation exposure that would result from isotopes with longer half-lives. Pure gamma emitters such as 99mTc or pure positron emitters such as 18F are preferred to reduce radiation exposure.^68–71^

## Conclusions

Some imaging techniques involve significant radiation exposure for ICU patients because of the critical patient conditions that require monitoring during hospitalization. Several possible best practices, both in the organizational and technical field and involving hospital administration, ordering physicians, radiographers, and radiologists, should be extensively implemented to reduce radiation exposure in ICU patients.
